# Rapid improvement in microaneurysms and stenosis of the visceral arteries in a patient with polyarteritis nodosa on follow-up angiography

**DOI:** 10.1093/rap/rkaa035

**Published:** 2020-07-23

**Authors:** Yoshinobu Matsuura, Hideaki Yakushiji, Yoshinori Katada

**Affiliations:** r1 Department of Rheumatology and Clinical Immunology, Saiseikai Senri Hospital; r2 Department of Critical Care Medical Center, Sakai City Medical Center; r3 Department of Respiratory Medicine and Clinical Immunology, Suita Municipal Hospital, Osaka, Japan

**Key message**

- Seven days of immunosuppressive therapy dramatically improved the angiographic findings of PAN.

Sir, Although aneurysms of the visceral arteries are one of the criteria for diagnosis of medium-sized arteritis, such as PAN, their clinical course is not well documented, because follow-up angiography is rarely performed. We present a patient who underwent angiography before and 7 days after receiving immunosuppressive therapy, showing marked improvement in stenosis, irregularities and microaneurysms of visceral arteries.

A 74-year-old Japanese man who presented with malaise and appetite loss for several days was admitted to a hospital. He had a known case of chronic obstructive pulmonary disease undergoing treatment with an inhaled muscarinic antagonist. Thirteen days after admission, he experienced retroperitoneal bleeding and was transferred to our hospital. On admission (day 1), he complained of back and abdominal pain and had a blood pressure of 150/90 mmHg, a pulse rate of 83 beats/min and a body temperature of 36.8°C.

Observed signs included upper abdominal tenderness without guarding and rebound tenderness. There were no palpable masses or signs of organomegaly, and dermal, muscular or neural abnormalities were absent.

Laboratory test results were as follows: white blood cell count, 22,960/µl; haemoglobin, 8.6 g/dl (baseline, 13.6 g/dl); platelet count, 360 000/µl; blood urea nitrogen, 37 mg/dl (baseline,18.3 mg/dl); serum creatinine, 1.6 mg/dl (baseline, 0.67 mg/dl); CRP, 16 mg/dl; ANA, 1/80; RF, 165 IU/ml; MPO-ANCA, negative; and PR3-ANCA, negative. Results of blood culture and antibodies for HBV, HCV and HIV were negative.

**Fig. 1:**
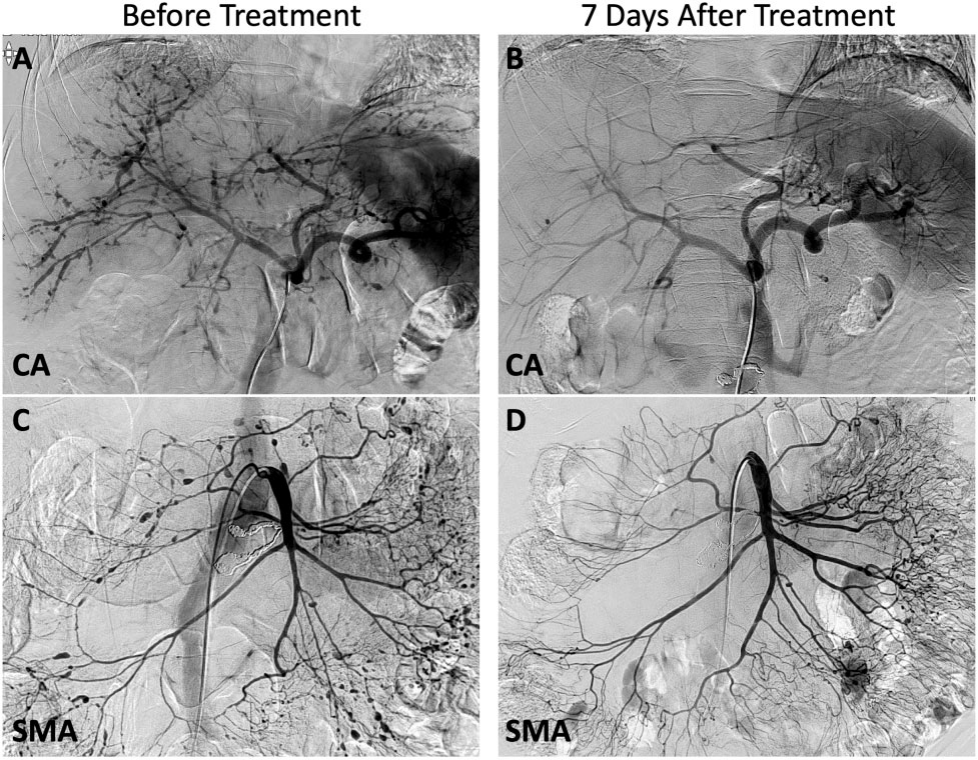
Angiographic findings before and after immunosuppressive therapy Angiographic findings in the CA and SMA, pre-treatment and 7 days after the immunosuppressive therapy. Microaneurysms and occlusive lesions, such as stenosis, irregularity and occlusion, were observed before treatment (**A**, **C**). Most of these findings disappeared 7 days after immunosuppressive therapy (**B**, **D**). CA: coeliac artery; SMA: superior mesenteric artery.

CT angiography of the abdomen revealed retroperitoneal bleeding, with several aneurysms in the branches of the coeliac artery and superior mesenteric artery, and conventional angiography revealed numerous stenotic lesions, lumen irregularities and microaneurysms (Fig. 1A and C). Subsequently, treatment was performed with coil embolization for the ruptured aneurysms in the anterior and posterior inferior pancreatico-duodenal arteries, which were considered to be the source of bleeding.

On day 7, after the diagnosis of PAN, treatment was initiated with 1 g i.v. of methylprednisolone for 3 days followed by 1 mg/kg of prednisolone, combined with biweekly 500 mg i.v. of CYC.

On day 13, after 7 days of immunosuppressive therapy, angiography was performed again to evaluate the therapeutic efficacy of the regimen, and dramatic improvements were observed in the visceral arteries (Fig. 1B and D). One year after the diagnosis, the patient remained asymptomatic with 5 mg/day prednisolone and AZA.

PAN belongs to a spectrum of necrotizing arteritis of medium-sized arteries that causes organ infarction and aneurysm formation. Approximately 50% of patients with systemic PAN have mild to severe gastrointestinal involvement [1], and aneurysms are serious complications [2]. The diagnosis of PAN is challenging because its symptoms are non-specific. Angiographic findings are especially important in cases without a suitable site for biopsy and form one of the 10 diagnostic criteria according to the ACR (1990). CT angiography is a useful alternative to conventional angiography because it is a less invasive procedure. However, conventional angiography is superior in detecting subtle areas of stenosis, occlusion or aneurysms in small arteries [3].

We performed a follow-up conventional angiography only 7 days after commencement of immunosuppressant therapy. A significant improvement was observed in a shorter time period than those reported in previous studies [4, 5]. We believe that this improvement is attributable to immunosuppressive treatment and is not the result of embolization, because embolization was performed only at two small peripheral arterial sites, whereas improvement of vascular lesions was observed throughout the coeliac and mesenteric arteries.

In 56 PAN patients who underwent angiography, occlusive lesions, such as lumen irregularity, stenosis or occlusion, were found in 55 (98%) patients, and aneurysms and ectasia occurred in 34 patients (61%), suggesting that occlusive lesions present as initial manifestations of the disease [6]. Concordantly, pathological changes usually emerge from the inner media and not from the adventitia, and aneurysm formation is thus a subsequent change attributable to PAN [7], with an associated risk of rupture. In our case, some microaneurysms remained, despite the elimination of most stenotic lesions with immunosuppressive therapy, indicating that occlusive lesions were more responsive to the treatment.

The early arterial stenotic lesions of PAN responded to immunosuppressant therapy sooner than expected. Therefore, early diagnosis and treatment are crucial for preventing aneurysm formation and rupture. Furthermore, early improvement of angiographic findings with immunosuppressive therapy can distinguish PAN from other diseases, such as lymphangioleiomyomatosis, bacterial infection and segmental arterial mediolysis, that can have similar angiographic findings.

*Funding*: No specific funding was received from any bodies in the public, commercial or not-for-profit sectors to carry out the work described in this article.

*Disclosure statement*: The authors have declared no conflicts of interest.
